# The Effect of Bug-in-Ear eCoaching on Pre-Service Teachers’ Implementation of Functional Communication Training

**DOI:** 10.3390/bs15070989

**Published:** 2025-07-21

**Authors:** Melih Çattık, Esra Orum-Çattık, Ahmet İlkhan Yetkin

**Affiliations:** 1 Research Institute for Individuals with Disabilities, Anadolu University, 26470 Eskişehir, Türkiye; 2 Special Education Department, Eskişehir Osmangazi University, 26040 Eskişehir, Türkiye; eocattik@ogu.edu.tr; 3 Special Education Department, İnönü University, 44210 Malatya, Türkiye; ahmetilkhan.yetkin@inonu.edu.tr

**Keywords:** bug-in-ear e-coaching, functional communication training, problem behaviors, communication skills, pre-service teachers, autism spectrum disorder

## Abstract

The study examined the effect of BIE eCoaching on the functional communication training (FCT) implementation skills of pre-service teachers (PTs) and the effect of FCT implemented by PTs on reducing problem behaviors and increasing communication skills of children with autism spectrum disorder (ASD). After the intervention, it was examined whether PTs and children with ASD maintained and generalized the acquired behaviors. Moreover, PTs answered a social validity form before and after the intervention. The methodology of this study involved single-subject research with a multiple-probe design between pairs of participants. The BIE eCoaching intervention was effective in children with ASD’s use, maintenance, and generalization of FCT. FCT was effective in reducing problem behaviors, increasing communication skills, and maintaining and generalizing these skills in children with ASD. Social validity findings showed that PTs’ perceptions of BIE eCoaching changed positively at the end of the intervention.

## 1. Introduction

Practitioners should use evidence-based practices during the training sessions for persons with disabilities (Odom, 2008). Effective feedback provided individually, regardless of the implementer, increases the impact of the practice (Rodgers et al., 2019; Scheeler et al., 2012). In the literature, it is noted that feedback is most effective when it is response-specific (Shute, 2008), targeted (Hattie & Timperley, 2007), and immediate (Coninx et al., 2013; Scheeler & Lee, 2002). Performance feedback, a practical component of coaching practices, is crucial in the education of pre-service teachers (PTs), particularly for implementing evidence-based practices with high implementation reliability (Fallon et al., 2015; Regan & Weiss, 2020). Performance feedback can be provided to the PTs in two forms (immediate or delayed) during coaching (M. L. Rock et al., 2014). Delayed feedback (Sharplin et al., 2016), which is based on the principle of giving feedback on the same day or after a few days following the monitoring of the implementation, has limitations, such as the person to whom feedback is given forgetting the feedback given, errors made during teaching cannot be prevented. However, there are also some situations where delayed feedback can be used. For example, Aljadeff-Abergel et al. (2017) state in their study that if the next performance opportunity is imminent, immediate feedback may be best; however, if there are several days between performance opportunities, it may be better to delay feedback. In other words, delayed feedback may be preferable, especially when the performance opportunity is delayed. This is because feedback has a precursor effect on performance, especially when the next performance opportunity is delayed. The closer this effect is to the next performance, the stronger it may be. In contrast, in immediate feedback, the coach can intervene during instruction and prevent implementation errors (Coninx et al., 2013). Both methods have proven to be useful for practice. Immediate feedback offers the opportunity to identify mistakes during implementation, reduce errors in subsequent implementations (Lemow et al., 2017), acquire a behavior or skill more quickly, and modify behaviors in a shorter timeframe (Artman-Meeker et al., 2017). In other words, instant feedback enhances the practitioner’s correct responses and provides a more qualified, rapid, and enduring learning experience (Regan & Weiss, 2020; Schaefer & Ottley, 2018). However, immediate feedback requires the physical presence of a supervisor, which can create limitations in terms of damage to the classroom climate (Scheeler et al., 2006), time (Scheeler et al., 2012), and cost (N. Rosenberg & Huntington, 2021).

While Bug-in-Ear (BIE), a type of coaching, used to be based on providing feedback to individuals through wireless headphones from behind an observation mirror, with the development of technology, it has become possible to virtually observe teaching and provide real-time feedback instead of having a supervisor physically present in the classroom (Ottley & Hanline, 2014). As such, it has taken its place among the implementations used by researchers as a type of eCoaching in recent years (Gander & Dann, 2023). In BIE eCoaching, a camera or a device with a camera connected to the internet in the training environment broadcasts the training sessions in real time, while a supervisor can watch the session instantly from a different location (Hoppe et al., 2024; Horn et al., 2021). The supervisor can provide positive and corrective feedback to the practitioner throughout the implementation (Regan & Weiss, 2020; Scheeler et al., 2010). Thus, with BIE eCoaching, practitioners can implement practices with positive learning experiences and high implementation fidelity (Horn et al., 2021; M. L. Rock et al., 2011).

In recent years, supporting individuals with ASD in inclusive educational environments from early childhood has become one of the primary goals of special education. However, training teachers who are qualified to respond to the complex and individualized needs of these individuals remains a significant problem. Studies conducted in Turkey have shown that prospective special education teachers have insufficient knowledge about ASD, which limits their ability to provide effective teaching practices (Rakap et al., 2016). PTs were found to have deficiencies in both theoretical knowledge and practical skills in establishing effective communication with individuals with ASD, managing problem behaviors, and providing individualized instruction. In another study conducted by Rakap et al. (2016), it was revealed that teacher candidates need more implementation-based training and structured feedback to be able to teach individuals with ASD effectively. It was emphasized that systematic support during implementation plays a critical role in increasing PTs’ pedagogical competence. This situation indicates that teacher training programs should not only focus on theoretical knowledge but also on high-quality implementation and mentoring processes. Additionally, a study conducted by Rakap (2017) found that systematic implementation support provided to PTs through technology—such as video analysis and digital feedback mechanisms—was practical in developing instructional skills. Such innovative approaches have been reported to not only improve PTs’ performance in classroom implementations but also positively influence their self-confidence and perceptions of professional competence. These findings highlight the need for professionals working with individuals with OSB to support their professional competence, not only through classroom observation but also through systematic, data-driven, and practice-oriented teaching strategies.

Time limitations and limited financial resources often limit supervisors’ ability to observe practice and provide feedback (Rodgers et al., 2019). For this reason, PTs mostly have clinical learning experiences when they start their professional lives (Horn et al., 2021). This situation affects professional satisfaction and interrupts critical learning experiences for students with disabilities. On the other hand, coaching is one of the most effective ways to offer PTs, especially for implementing highly reliable, evidence-based practices (Kretlow & Bartholomew, 2010). The BIE eCoaching enables more practitioners to receive feedback in a shorter timeframe and at a lower cost (Gander & Dann, 2023; M. L. Rock et al., 2014). The practitioner can practice repeatedly while receiving real-time performance-based feedback (Horn et al., 2021; M. L. Rock et al., 2009).

Studies conducted with PTs emphasize that BIE practice is very effective in seeing how much of the theoretical knowledge learned can be reflected in practice (Coogle et al., 2017; Rakap, 2017). In addition, BIE prevents practitioners from missing learning opportunities that may be critical for students and practitioners during their work with students and may not be apparent enough to correct after implementation. Even if the student makes proper eye contact, the teacher may not notice or reinforce it, making it difficult for the positive behavior to be repeated. However, with BIE, the teacher receives an instant “Great eye contact! Please reinforce” alert from the coach via earpiece. This allows the teacher to acknowledge the student immediately. Such instant interventions enable students to learn target behaviors much faster.

Current studies conducted with BIE show that teachers and PTs who use this technology coaching increase the level and frequency of using evidence-based practices and decrease the use of less effective practices (Hollett et al., 2017; M. L. Rock et al., 2014; Scheeler et al., 2010). They also stated that PTs improved their self-efficacy and decreased their anxiety levels after receiving performance feedback with BIE technology (Artman-Meeker et al., 2017; Rakap, 2017). BIE is a practical tool to improve teaching practice and enable PTs to use evidence-based practices correctly and effectively to eliminate the gap between research and practice, which has been frequently mentioned in special education in recent years (Scheeler et al., 2010). PTs show that immediate feedback provided through BIE is much more effective on teacher behavior than traditional feedback (delayed feedback provided face-to-face after the implementation) and that they improve their communication strategies through coaching sessions (Coogle et al., 2017).

When the current studies with BIE eCoaching are examined, it is seen that participants successfully learned practices such as embedded communication strategies (e.g., Coogle et al., 2017; Ottley et al., 2017), discrete trials training (McKinney & Vasquez III, 2014), and reinforcement (Scheeler et al., 2016). In addition, participants stated that they were satisfied with the coaching offered with BIE (Artman-Meeker et al., 2017), that real-time correction of their errors was effective in their learning (Scheeler et al., 2010), that their self-efficacy increased and anxiety levels decreased with the encouraging feedback they received (Coninx et al., 2013; M. L. Rock et al., 2014), and that they would recommend BIE to their colleagues (Scheeler et al., 2010). A growing body of research supports the use of BIE coaching in supporting teachers’ professional development (Israel et al., 2013; Schaefer & Ottley, 2018). Therefore, in recent years, research has suggested that BIE should play a more significant role in preparing PTs (McLeod et al., 2024; Rivera et al., 2024).

Functional communication training (FCT) is an evidence-based intervention that reduces problem behaviors by teaching individuals to use acceptable communication responses that serve the same or a similar function as the problem behavior (Carr & Durand, 1985; Ghaemmaghami et al., 2021). That is, the “functional” component refers to identifying the specific purpose of the problem behavior—such as obtaining attention, escaping a demand, or accessing a tangible item—and replacing it with an alternative communication behavior that meets the same need, thereby making it an adequate substitute. As in traditional supervision, addressing problem behaviors after they occur may not be effective, especially if there is a significant delay between the behavior and the intervention. In the context of FCT, alternative communication responses must be prompted and reinforced before or immediately following the antecedent events that typically evoke problem behaviors—rather than as a delayed reaction to those behaviors (McCambridge et al., 2014). In this case, BIE eCoaching is a perfect approach. In the literature, there is a minimal number of studies examining the level of FCT implementation of behavior analyst candidates (Artman-Meeker et al., 2017), paraprofessionals (N. E. Rosenberg et al., 2020), and their parents (Suess et al., 2014; Wacker et al., 2013). using BIE. However, there was no study in which FCT was provided to PTs. It is believed that substantial evidence is necessary to investigate the effects of coaching practices provided by BIE on coping with problem behaviors.

Previous studies have demonstrated that functional communication training (FCT) can effectively reduce challenging behaviors and teach alternative communication behaviors (Gregori et al., 2022). However, concerns persist regarding the maintenance and generalization of these skills, particularly when implemented by PT’s or parents (Storie et al., 2017). Similarly, the long-term effects of real-time feedback on the maintenance of teacher and student performance over time are less clear (Artman-Meeker et al., 2017). Therefore, in this study, we examined the effect of BIE eCoaching on the use of FCT intervention by pre-service special education teachers. Additionally, the effect of FCT applied by PTs on reducing problem behaviors and enhancing the communication skills of children with ASD was examined. Finally, we searched the social validity of the study in terms of PTs.

1. Is the BIE eCoaching implementation, which aims to equip PTs with the skills to implement FCT, effective in enabling PTs to
  1a Effectively implement the steps of FCT at the implementation level?
  1b Are they effective in enabling PTs to demonstrate the steps of FCT implementation after a period of time?
  1c Are they effective in enabling PTs to demonstrate the steps of FCT implementation in different settings?
2. Does the FCT implementation provided by PTs improve the following aspects of children with ASD taught by PTs?
  2a Reducing the level of problem behaviors?
  2b Acquiring functional communication skills?
  2c Maintaining the acquired behaviors at a similar level after a period of time following the implementation?
  2d Are they effective in enabling them to demonstrate the acquired behaviors in different environments and with different individuals?
3. What are the views of the PTs participating in the study regarding the social validity of the study before and after the implementation?

## 2. Methods

### 2.1. Participants

Before starting the study, the researchers obtained ethics committee approval from the Social and Human Sciences Ethics Committee of a university in Türkiye (No.: E-54380310-050.99-308196 and 26 April 2022). The participants of this study were three PTs and three children diagnosed with ASD in early childhood. The PTs are undergraduate students in the Department of Special Education at a university in Türkiye. The PTs had completed the Applied Behavior Analysis (ABA) course during their undergraduate education, before the research, and participated in the study voluntarily. We obtained written informed consent from the PTs.

The children participating in the study receive group education from the developmental support unit at the same university two days a week. We looked for participant children to (a) be diagnosed with ASD, (b) be between 36 and 72 months of age, (c) follow instructions of at least two words, (d) pay attention to auditory stimuli for at least one minute, (e) can make choices, and (f) exhibit at least one problem behavior due to limitations in communication skills. Whether children with ASD met the requirements was evaluated by examining the reports, interviewing the teachers and parents of the children receiving group instruction, and making observations. In addition, we administered the Gilliam Autistic Rating Scale-2 Turkish version (GARS-2 TV), Anadolu Sak Intelligence Scale (ASIS), Gazi Early Childhood Assessment Tool (GECAT), and Test of Early Language Development—Third Edition: Turkish (TELD3: T) to children with ASD. The study’s second author conducted the assessments of the GARS-2 and the GECAT. Two separate experts, qualified to evaluate the related instruments, conducted the ASIS and the TELD3:T. We obtained written informed consent from the parents of the children who met the eligibility requirements to participate in the study. We matched the PTs and children as pairs after they were identified. To determine each child’s baseline communication profile, two assessment tools were administered. The GECAT was used to evaluate functional communication abilities in everyday contexts, providing insights into the children’s spontaneous expressive and receptive skills. In addition, the TELD-3 was administered to obtain standardized scores for expressive and receptive language development. The combined use of these tools allowed for a more comprehensive understanding of each participant’s communication strengths and needs, both in functional and standardized terms.

The coach and other researchers involved in the study had no authority to supervise the PTs. Similarly, the PTs had no close relationship with the children with ASD or their families.

Dyad 1: PT1-C1 is a 20-year-old third-year undergraduate student in the Department of Special Education. She had no formal training in FCT beyond an applied behavior analysis course. C1 is a 48-month-old boy diagnosed with ASD who had been attending the university’s developmental support unit for approximately 15 months at the time of the study. He uses two-word utterances but shows significant delays in both expressive and receptive language. According to the GARS-2, his total score was 80, indicating moderate symptoms of ASD. His developmental profile revealed substantial delays across multiple domains: psychomotor development (PMD = 60), cognitive development (CD = 45), language development (LD = 36), and social-emotional development (SED = 45). The TELD-3 results indicated that both his receptive and expressive language skills were below those expected for a 1.3-year-old. C1 mostly holds the adult’s hand to reach the desired object or activity or attempts to reach the object physically. However, when their requests are not met, they show intense problem behaviors. C1 exhibited frequent problem behaviors, including hitting, shouting, and crying. These behaviors typically occurred during transitions, group instruction, play routines, and mealtimes—often when he was presented with demands, denied access to preferred items, or faced changes in routine. Due to his limited communication abilities, C1 was identified as an appropriate candidate for FCT to support the development of alternative communication strategies that serve the same function as his problem behaviors.

Dyad 2: PT2 is a 21-year-old second-year undergraduate student enrolled in the Special Education department. He has not received systematic training in FCT and has only acquired basic knowledge through a course in applied behavior analysis. C2 is a 42-month-old boy diagnosed with ASD who has been receiving education at a university-affiliated developmental support unit for approximately 10 months. He can form two- to three-word phrases but exhibits significant delays in both receptive and expressive language development. His GARS-2 total score is 80, and his ASIS score is 60. According to the GARS results, his psychomotor development score is 70, his cognitive development score is 60, his language development score is 52, and his social-emotional development score is 40. In the TELD-3 assessment, both receptive and expressive language ages were determined to be below 1.3 years. C2 frequently exhibits problem behaviors, such as throwing himself to the floor, hitting himself, and crying during transitions, tabletop tasks, mealtimes, and challenging activities to avoid undesirable tasks. Most of these behaviors occur in situations of task refusal, demand overload, or inability to reach the desired object. Due to limited communication skills and significant behavioral issues, C2 was deemed an appropriate participant for FCT intervention.

Dyad 3: PT3 is a 26-year-old female teacher candidate in her third year of the Special Education program. She has not received systematic training in FCT, having only completed a course in applied behavior analysis. C3 is a 61-month-old boy diagnosed with ASD who has been receiving educational support for approximately 27 months. He typically uses single-word utterances and rarely forms two-word structures. His language and cognitive development are significantly limited. His GARS-2 total score is 88, and his ASIS score is 57. According to GARS results, his psychomotor development score is 58, cognitive development score is 43, language development score is 40, and social-emotional development score is 42. In the TELD-3 assessment, both receptive and expressive language ages were determined to be below 1.3 years. C3 frequently exhibits problem behaviors during classroom routines and transitions. These behaviors include hitting oneself and others and biting one’s hands. These behaviors typically manifest as avoidance of unwanted activities, resistance to changes in routines, and a tendency to react to uncertain situations. Most of these behaviors have been observed in response to resistance to requested activities, uncertainty, or changes in the environmental order. The significant delay in language skills and the functional nature of the behaviors make C3 an appropriate candidate for FCT intervention.

Coach. The coach holds a doctorate in special education. He has nearly 20 years of experience working with children with ASD in early childhood and is a faculty member at the same university. He has scientific studies and research projects on coaching practices in the education of children with ASD. He conducted all the coaching processes for the PTs who participated in this study.

Observer. A research assistant doctor with a doctorate in special education collected the inter-observer agreement and implementation of fidelity data. Before collecting the data, the observer received information and training on the coaching process.

### 2.2. Settings and Materials

The participant pairs conducted the baseline, implementation, and follow-up sessions at the home of the children with ASD. The coach monitored the implementation of the participant pairs from her own home. Care was taken to ensure the room where the participant pairs carried out the activity was free from distracting stimuli. The coach and the participant pairs closed the door so no other person could enter the room during the intervention. Generalization sessions were conducted in the park, in the garden of children with ASD (C1 and C2), and in the teacher’s (C3) home, where the children received individualized education. The coach used a laptop (MacBook Air 13″) with a camera, and the PT used an iPad (Apple 10.9″). The coach monitored the implementation and provided coaching through the ZoomTM Pro application installed on both devices. Both devices were connected to two separate secure wireless networks. The PTs used a Xiaomi Buds 4 Active headset provided by the researchers during all sessions. Additionally, during implementation, all sessions were recorded on the cloud storage associated with the ZoomTM Pro account. The materials used by the PTs during the activities included coloring, puzzles, playground equipment, playdough, sorting picture cards, game materials, and reinforcers. The problem behavior assessment forms, problem behavior data recording forms, communication skills data recording forms, and implementation fidelity data recording forms were adapted from the study by Pektaş Karabekir (2021) and prepared by the coach for this research.

### 2.3. Dependent and Independent Variables

The dependent variable for PTs was the level of implementation of FCT steps. The dependent variable for child participants was the exhibition of functional communication behaviors (communicating appropriately in a manner that another person can understand) and problem behaviors. The independent variable for the PTs in the study was the eCoaching with BIE used to acquire FCT behaviors. The independent variable for the child participants was the FCT used by the PTs. The FCT steps are (a) identifying problem behavior, (b) conducting a functional behavior assessment, (c) determining the alternative communication skill, (d) teaching the new communication skill, and (e) ensuring generalization and maintenance (O’Brien et al., 2021).

### 2.4. Research Model

The study’s model is single-subject research with a multiple-probe design and probe trials across dyads. The experimental control was ensured by the fact that there was no change in the baseline level; there was a gradual increase in the level of targeted skills for the participant pairs when eCoaching with BIE was implemented; there was no change in the data of the participant pairs where eCoaching with BIE was not implemented, and similar changes were observed in the data of other participant pairs as the implementation took place (Gast et al., 2018). We preferred this model for reasons such as the necessity of not collecting continuous baseline data due to the study on problem behaviors of children with ASD, more accessible data collection, controlling maturation and testing, and not requiring the withdrawal of effective practice.

### 2.5. Procedure

#### 2.5.1. Coach’s Functional Behavioral Assessment

Before implementation began, the coach used the functional behavior analysis process to determine the functions of the behaviors exhibited by children with ASD. In this process, the stages of defining the problem behavior, determining the stimuli that precede the behavior, determining the stimuli that occur during or after the behavior, identifying the environmental variables that cause the problem behavior, and testing hypotheses about the cause of the problem behavior were included. Coach conducted interviews with parents and teachers of children with ASD to obtain information about problem behaviors and communication skills. The interviews asked questions about children’s general behaviors, communication skills, and environmental events related to the emergence and maintenance of problem behaviors. The interview form consisted of seven open-ended questions prepared by the researchers based on the literature and finalized after receiving the opinions of three field experts. The first researcher recorded and transcribed interviews with the written consent of the interviewees.

The coach conducted observations, interviews, and trial-based functional analyses to determine the functions of problem behaviors and communication skills (Lambert et al., 2012). The coach observed children with ASD during group sessions, play activities, meal times, and transition activities in the unit where they received training. Observation sessions lasted 45 min each for the children. The information obtained from the observation sessions was recorded using the Antecedent Behavior Consequence recording form prepared by the researchers based on the literature. As a result of the observations, the problem behaviors exhibited by children with ASD were identified.

**Figure 1:**
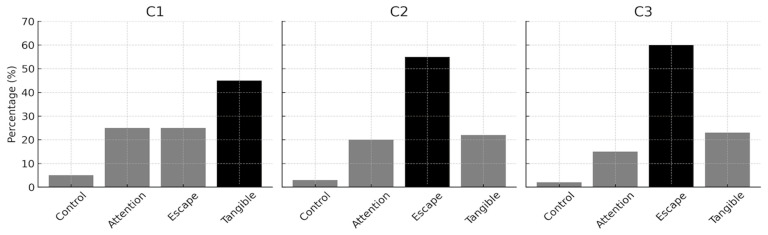
Percentage of problem behavior by conditions for each child’s trial-based functional analysis.

Using the data obtained from interviews and observations, Coach conducted functional analysis of other behaviors (FAOB) sessions with each of the children with ASD. In this process, she organized multi-stimulus preference assessment sessions and FAOB sessions. In the multiple stimulus preference assessment sessions, the coach recorded the different object/activity/food preferences of children with ASD on the multiple stimulus assessment form prepared by the researchers based on the literature. The multiple-stimulus preference assessment sessions were organized into three sessions for each child. At the end of the sessions, the most preferred, moderately preferred, and least preferred stimuli were determined. In the FAOB sessions, the coach determined the functions of problem behaviors by exposing children with ASD to situations that were thought to cause problem behaviors based on the information obtained from the interview and observation sessions. In this process, three sessions were held with each child to determine the functions of behaviors (“obtaining” or “escaping”). The findings were recorded using the trial-based functional analysis recording form prepared by the researchers based on the literature (Lambert et al., 2012; Larkin et al., 2016). According to the results of the FAOB, C1 exhibited problem behaviors mostly to obtain attention, while C2 and C3 exhibited problem behaviors mostly to escape from the activity. Finally, the coach decided on functional communication skills with the parents and teachers of children with ASD. The results of the trial-based functional analysis are shown in Figure 1.

#### 2.5.2. Coach Informing the Pre-Service Teachers About the Technology Setup

The coach informed the PTs about using the Bluetooth headset and making voice calls. The coach and the PTs then conducted trial sessions in the environments where the implementation would be carried out regarding the setup of the devices, connection control, and the realization of the eCoaching session.

#### 2.5.3. Baseline

The coach conducted baseline sessions of the PT independently with all participant pairs for three sessions before the implementation. The coach and the participant pair were in the same environment and were only in the baseline sessions. The coach conducted all other sessions online. During the baseline sessions, the coach kept the objects, foods, and activities that the children preferred the most in the environment and did not provide any information to the PT about these materials. The coach provided instructions to the PT and did not intervene. The sessions were recorded on a video camera. After obtaining stable baseline data, eCoaching was started with the first pair of participants. While the intervention was ongoing with the first participant pair, baseline data were collected from the other pairs at regular intervals. After the intervention was completed with the first participant pair, baseline data were collected with the second participant pair until stable data were obtained, and baseline data were collected periodically with the third participant pair. After the intervention was completed with the second participant pair, baseline data were collected with the third participant pair until stable data were obtained. The data obtained were recorded using the intervention fidelity data recording form. The coach collected the baseline data on problem behaviors and communication skills of children with ASD in three consecutive sessions. The data were marked on the problem behavior and communication skills data recording forms.

#### 2.5.4. Pre-Service Teacher Training Sessions

After the baseline sessions, the coach conducted training sessions with the PTs via Zoom^TM^. In these sessions, the coach provided an orienting presentation to the PTs about the problem behavior and target behavior, including recording the behavior, using prompts, reinforcement, the time-delay procedure, and the steps of FCT. After the presentation, three videos were shown to the PTs, in which the coach acted as a teacher and the second researcher acted as a student, including the FCT steps. The PTs were asked to record the behaviors in the videos on the data recording forms sent to them. The second researcher acted as a student and asked the PTs to have a trial session with him/her regarding the implementation steps of the FCT. During the trial session, the coach provided encouraging and corrective feedback to the PTs (M. L. Rock et al., 2014). After the PTs completed their training, the implementation started.

#### 2.5.5. BIE Intervention

Implementation sessions were conducted simultaneously with each participant pair in the homes of the participant children between 10:30 and 11:30 a.m. on five weekdays. The implementation sessions were organized as one-to-one instruction. During the implementation, the coach and the PT wore Bluetooth headsets. The coach met in his office, and the pair of participants met in the homes of children with ASD. During the sessions, the PT placed the iPad where the implementation could be easily seen. The PT connected the Bluetooth headset to the iPad, opened the Zoom^TM^ application, and informed the coach that she was ready. The coach kept his camera off during the sessions to avoid distracting the pair of participants. Only the PT heard the coach’s feedback via Bluetooth headset. The coach waited for a while; the PT used the steps and provided corrective feedback when she did not perform the steps correctly, and provided encouraging feedback when she did.

Even if the PTs implemented the FCT with 100% accuracy, the implementation sessions continued until 100% performance was obtained in children’s communication skills. After the criterion was met with the first pair of participants, the process continued with the other pair. During implementation, the PTs used a time-delay procedure and collected data on children’s problem behaviors and communication skills. In the implementation sessions, the first session consisted of 0-s trials, followed by 4-s trials. During the trials, the coach recorded the PT’s behaviors on the fidelity of the implementation data recording form. He carefully observed all the PTs’ reactions and provided immediate positive feedback (e.g., “You got him/her interested in the object in the appropriate time, great!”) and/or corrective feedback (e.g., “You need to reinforce here”) during the practice. At the end of the implementation sessions, the coach provided a brief evaluation of the PT’s performance and answered any questions they may have had. The frequency and number of immediate feedback provided by the coach during the implementation sessions gradually decreased. The feedback was as clear and explicit as possible to avoid distracting the PT. The coach recorded the PTs’ behaviors on the implementation fidelity data recording form. During the implementation sessions, the coach recorded the PT behaviors on the implementation fidelity data recording form (Appendix A: FCT implementation fidelity data recording form sessions consisting of trials with 0 s/4 s of delay time), and the reactions of the children with ASD were recorded on the problem behavior data recording form and the communication skills data recording form. The PTs also recorded the reactions of children with ASD. The FCT steps are implemented by the PTs in the practice sessions.

#### 2.5.6. Maintenance

The coach included maintenance sessions after obtaining stable data in the intervention sessions. He collected follow-up data from the participant pairs simultaneously: 1, 3, 5, and 7 weeks after the last intervention session with the first participant and 1, 3, and 5 weeks after the last session with the second and third participants. The maintenance sessions were conducted in the same way as the baseline sessions. The data obtained from the PTs were marked on the implementation fidelity data recording form, and the data obtained from the students (both by the PTs and the coach) were marked on the problem behavior and communication skills data recording forms.

#### 2.5.7. Generalization

Generalization sessions were conducted with participant pairs as pre-test and post-test sessions. The generalization pre-test sessions were conducted immediately after the baseline and post-test sessions, after stable data were obtained in the intervention sessions. Generalization sessions were conducted in the playground of the children’s homes with ASD (C1 and C2) and at the home of their teacher (C3), where they received individualized education. In the generalization sessions, the process followed in the baseline sessions was followed. The data obtained from the PTs were marked on the implementation fidelity data recording form, and the data obtained from the students (both by the PTs and the coach) were marked on the problem behavior and communication skills data recording forms.

#### 2.5.8. Social Validity

We collected social validity data from the PTs at the end of the study and the implementation sessions. Two 5-point Likert-type questionnaires were prepared for FCT and the coaching process to collect social validity data. The questionnaires comprised five items, each rated on a scale from 1 (strongly disagree) to 5 (strongly agree). The questionnaire items were checked by experts who practice in special education for suitability. Adaptations were made to the items based on feedback from the experts.

### 2.6. Data Analysis

The effectiveness of the data collected from children in the study was analyzed through visual analysis and effect size calculations (Kratochwill et al., 2010). The data collected to evaluate the effectiveness of the FCT implementation presented by PTs on reducing problem behaviors in children and imparting appropriate communication skills to children were plotted on a line graph. The data plotted on the graph were visually analyzed in terms of trend, level, consistency, and immediate effect. To analyze the data obtained in the study in terms of the immediate effect, the average of the last three data points in the starting level phase was calculated, as was the average of the first three data points in the implementation phase. The difference between these two averages was then calculated. The response percentage related to the children’s targeted problem behaviors and the correct response percentage related to their communication skills were calculated using the formula “(Correct Response Count/Total Response Count) × 100” (Gast et al., 2018). The response percentages calculated for the children’s sessions were plotted on a graph, and the effectiveness of the plotted data was evaluated. The follow-up data collected to evaluate the level of communication skills and problem behaviors exhibited by the children after a period of time following the implementation sessions, and the generalization data collected to evaluate whether the children were able to generalize the communication skills they learned, were also plotted on a line graph. The data plotted on the graph were analyzed visually. Tau-U (Start Level Trend Controlled) was also used to calculate the effect size of the data collected from the children in the study. Tau-U is an effect size calculation method that controls the baseline trend and determines the percentage of data points that do not overlap and show progress by comparing data points in the baseline phase with those in the implementation phase (Parker et al., 2011; Rakap, 2015). Additionally, Tau-U is an effective calculation method for demonstrating the rate of bidirectional change in socially essential behaviors that are targeted for both increase and decrease, as these behaviors are socially significant and are aimed to be acquired by children in research (Parker & Vannest, 2012). The values obtained from the Tau-U calculation range from 0 to 1. In this calculation method, values of 0.80 and above indicate “huge effect,” values between 0.60 and 0.80 indicate “large effect,” values between 0.20 and 0.60 indicate “moderate effect,” and values of 0.20 and below indicate “small effect.” (Parker et al., 2011; Vannest & Ninci, 2015). In this study, an online interface program was used to calculate Tau-U (http://www.singlecaseresearch.org/calculators/Tau-U accessed on 15 february 2023). The generalization data obtained were marked on the line graph. Implementation fidelity and inter-observer agreement data were calculated using the relevant formulas, and the corresponding percentages were reported. Social validity data were analyzed descriptively and shown in the table.

#### Implementation Fidelity and Inter-Observer Agreement

A research assistant with a PhD degree in special education collected the implementation of fidelity and inter-observer agreement data. Implementation of fidelity data was collected from 30% of each session of the coaching training and implementation (providing immediate feedback) sessions offered to the PTs. Implementation of fidelity data was calculated using the formula “observed practitioner behavior/planned practitioner behavior × 100”. When implementation fidelity data were available, the lowest value for BIE eCoaching training sessions was 99% (range, 98–100%), and the highest value was 100%. For sessions providing immediate feedback, the lowest value was 95% (range, 83–100%), and the highest value was 99% (range, 96–100%).

Interobserver agreement data regarding the PTs’ FCT implementation, communication behaviors of individuals with ASD, and problem behaviors were collected from 30% of each session. Interobserver agreement data were calculated using the formula “agreement/agreement + disagreement × 100.” Inter-observer agreement data for PTs in FCT practice at the baseline level were 89.3%, during intervention, 89.6%, during maintenance, 97.6%, and during generalization, 93.3%. Communication skills of children with ASD were, on average, 92.6% at the initial level, 94.6% during intervention, 95.3% during maintenance, and 95.3% during generalization. Problem behaviors of children with ASD were, on average, 91% at the initial level, 95% during intervention, 95% during maintenance, and 95% during generalization.

## 3. Results

The overall results of the study showed a significant increase in the behaviors of both PTs and children with ASD between baseline, implementation, and maintenance data. The PTs and children with ASD could generalize the acquired skills. The social validity findings obtained from the PTs and parents showed satisfactory behavior before and after the study.

### 3.1. Research Question 1

PT1 was unable to correctly implement FCT steps during the pre-intervention period (average 0%) but showed significant improvement in this skill after the BIE coaching process. The accuracy rate increased to an average of 76.3% during the implementation phase, and a level change analysis revealed a 50% improvement. The effect size was calculated as 45% using the Tau-U analysis (45% CI [23, 1]). One of the most striking findings is that PT1 continued to perform with an average accuracy of 93.75% in the retention phases (weeks 1, 3, 5, and 7). In generalization sessions, it was observed that they were able to transfer this skill to different conditions with 100% accuracy. These results demonstrate that PT1 not only acquired the skill with short-term coaching support but also sustained it over time and transferred it to different contexts. This highlights the potential of models incorporating direct feedback (e.g., BIE coaching) for effectively teaching structural interventions, such as FCT, to teacher candidates.

PT2 initially applied the FCT steps with an average accuracy of 8.9%. Following the coaching process, the rate increased to 75%, representing a 37.5% rise. The effect size was calculated as 45% (45% CI [23, 1]). The high accuracy rate of 95.8% in the retention sessions is noteworthy. Similarly, 100% accuracy was achieved in generalization sessions. PT2’s development demonstrates that a teacher candidate with only basic ABA training can effectively implement FCT with short-term support. This result supports the notion that the coaching process is not only an instructional tool for teacher candidates but also a lasting learning process.

PT3 achieved an average accuracy of 1.5% at the initial level, which increased to 70% after intervention. However, the level change remained limited at 17%. The Tau-U value was calculated as 53% (95% CI [19, 1]), indicating a moderate effect size. Despite this, PT3 achieved 100% accuracy in the retention sessions, demonstrating that learning was consolidated over time. Additionally, it achieved 100% accuracy in the generalization post-test.PT3’s lower performance during the intervention suggests that individual learning speeds and prior knowledge levels may influence FCT implementations. However, considering the retention and generalization data, the long-term benefits of the BIE coaching intervention are clearly evident for PT3 as well.

**Figure 2:**
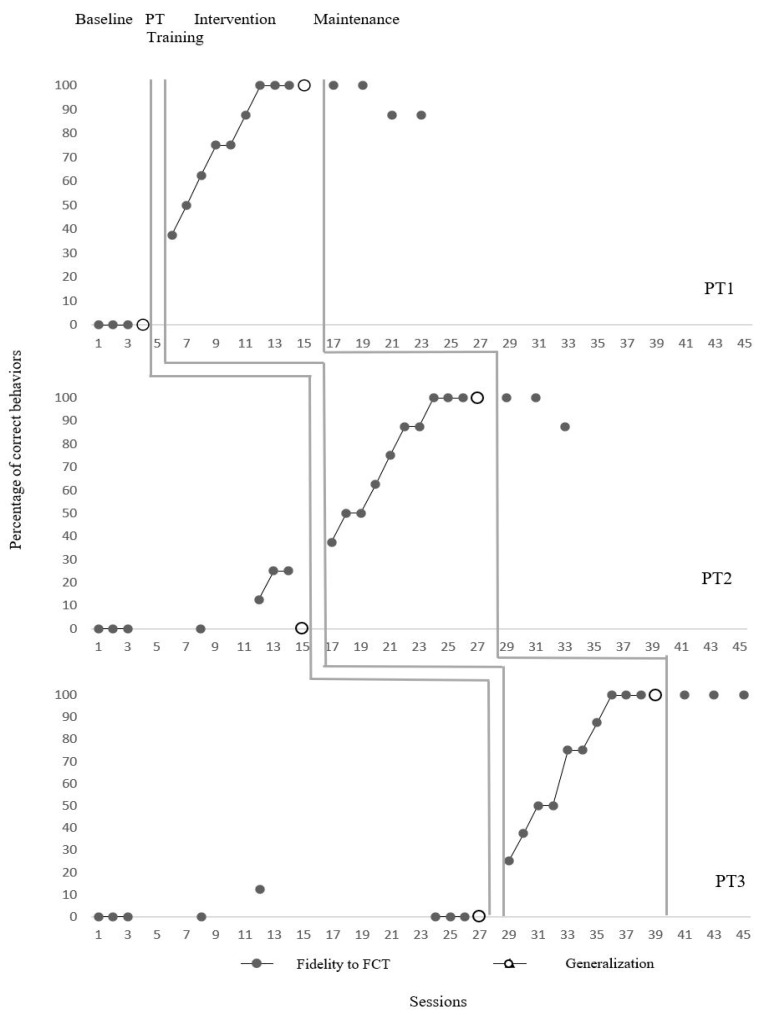
Pre-service teachers’ level of FCT implementation.

Although the initial levels of all three teacher candidates were quite low, significant increases were observed in both their implementation accuracy and retention-generalization performance following the BIE coaching process. These findings demonstrate that basic ABA training provided at the university level, when supplemented with structured coaching support, enables teacher candidates to learn and sustain complex implementations effectively. Additionally, alongside effect size calculations in single-subject studies, the sustainability of practitioner performance and generalizability provide strong evidence for the practical applicability of the intervention. Figure 2 shows the FCT data of PTs.

### 3.2. Research Question 2

C1 had never demonstrated the ability to request a target before the intervention (0%) but showed a significant increase in this skill during the intervention process. The ability to request was demonstrated with an average accuracy of 75% (range: 40–100%) during the implementation sessions. A 50% increase was observed between the two phases in the immediate effect analysis of level change. The effect size, according to the Tau-U value, is 34% (CI: 28–100%). In the maintenance sessions, this skill was sustained with an average of 95%, and the accuracy, which was 0% in the pre-test, increased to 100% in the post-test. When problem behavior data were examined, a high rate of 96.6% (range: 90–100%) was observed at the initial level. With the intervention process, there was a significant decrease in both the trend and the level, and this rate decreased to 35.5% (range: 0–80%) in the implementation sessions. According to the immediate effect analysis, the level difference was 33.3%, and the effect size was also 34% (CI: 28–100%). In the maintenance sessions, the problem behavior rate dropped to 0%, and it was eliminated (0%) in the generalization test. These findings suggest that C1’s limited communication skills can be functionally improved through FCT, which may lead to a sustained reduction in problem behaviors.

C2 initially demonstrated the skill of requesting a target with only 4.2% accuracy. During intervention sessions, participants were able to use this skill with an average accuracy of 76% (range: 40–100%), and the level change was calculated to be 46.7%. According to the Tau-U analysis, the effect size was 51% (CI: 20–100%). In maintenance sessions, the skill was sustained with 96.6% accuracy, and performance in the generalization pre-test, which was 0%, increased to 100% in the post-test. The problem behavior rate for C2 was initially an average of 94.2% (range: 90–100%), which decreased to 31% (range: 0–70%) during the intervention process. The level of change was calculated as 36.6%, and the effect size was 51% (CI: 20–100%). During the maintenance phase and the final generalization test, problem behaviors were eliminated (0%). These results demonstrate that FCT not only enhances communication skills but also enables children to reduce problem behaviors by interacting with their environment in more appropriate ways.

C3 initially demonstrated target request ability with an average accuracy of 5%. With intervention sessions, the rate increased to 87% (range: 0–80%), representing a 33.3% difference in level. According to the Tau-U analysis, the effect size was 53% (CI: 19–100%). No request skills were observed in the maintenance data (0%), suggesting difficulties in sustaining the skill. However, the skill was demonstrated with 100% accuracy in the generalization test. Problem behaviors in C3 were initially observed at a rate of 95% (range: 90–100%), which decreased to 25% (range: 0–80%) after the intervention. The effect size was again 53% (CI: 19–100%). Problem behaviors completely disappeared in both the maintenance and generalization processes (0%). The observed deficiency in skill maintenance in C3 highlights the importance of post-instructional follow-up implementations, while generalization success supports the transferability of FCT to different environments.

**Figure 3:**
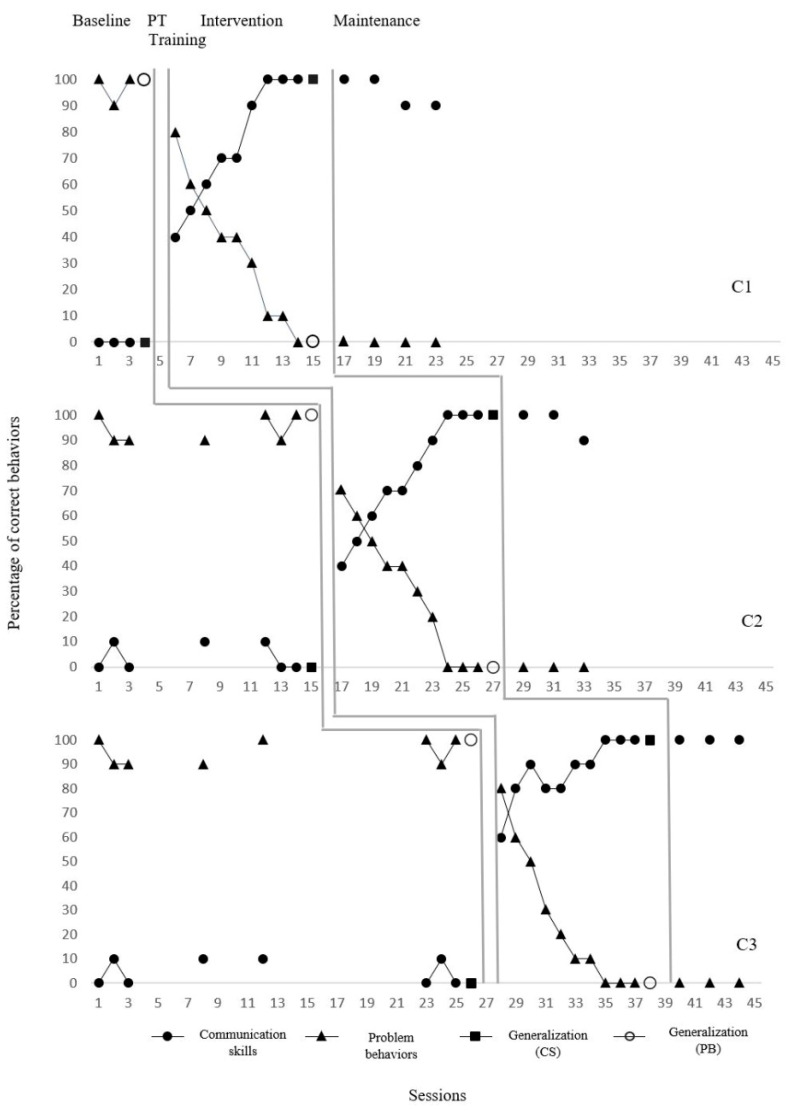
Communication skills and problem behavior levels of children with ASD.

All three children have extremely limited communication skills at the beginner level and high rates of problem behavior. With the intervention process, target skills have been acquired, generalized, and largely sustained. At the same time, there has been a significant decrease in problem behaviors, and in some cases, they have entirely disappeared. These findings demonstrate that structured FCT instruction for teacher candidates can have powerful and positive effects not only on teacher behavior but also directly on child outcomes. Data on communication skills and problem behaviors of children with ASD are shown in Figure 3.

### 3.3. Research Question 3

The second researcher collected the social validity data from the PTs face-to-face. All PTs completed the questionnaire. The social validity results demonstrate a clear and consistent improvement in PT’s perceptions of BIE coaching following the intervention. Across all five items, mean scores increased substantially from pre- to post-intervention, indicating enhanced acceptability, feasibility, and preference for BIE. The most dramatic shifts were observed in participants’ self-efficacy in learning to use an intervention independently (M increased from 2.3 to 5) and in their preference for BIE over traditional face-to-face coaching (M increased from 1.3 to 5), suggesting that the real-time and individualized nature of BIE may foster a greater sense of competence and autonomy.

All participants consistently rated immediate feedback as more helpful than delayed feedback, both before and after the intervention, reinforcing the value of timely guidance in skill acquisition. While perceptions of cost and time efficiency varied slightly across individuals, the overall trend favored BIE as a more efficient alternative. These patterns suggest that BIE coaching was not only effective in improving implementation fidelity but was also perceived as a practical and scalable professional development method by the participants. The findings regarding the social validity data collected from PTs are shown in Table 1.

## 4. Discussion

In this study, we examined how the BIE eCoaching method can support PTs’ FCT implementation and the effects of this instruction on the communicative and problem behaviors of children with ASD. This is not only the teaching of a technical skill, but also a process through which sustainable and correct practices are ensured among participants through coaching. This demonstrates that BIE can serve as a scalable and feasible monitoring tool for implementation that typically requires face-to-face support (Artman-Meeker et al., 2017). Additionally, the ability of teacher candidates to sustain and generalize the skills they acquire supports the long-term feasibility of BIE-supported coaching implementation in school-based teacher education settings where traditional monitoring is limited (N. E. Rosenberg et al., 2020).

Beyond teacher outcomes, this study also demonstrated that FCT implementation led by PTs supported through BIE can lead to meaningful improvements in children’s behavior. Decreases in problem behaviors and increases in communication attempts were consistently observed across all participants. These gains were not only sustained over time but also generalized to new settings. Providing coaching to PTs through BIE may indirectly benefit children. This is a significant contribution, as the vast majority of previous BIE research has focused solely on teacher behavior and has not included child-level outcomes (Coogle et al., 2017; Scheeler et al., 2012). Therefore, this study, which examines both teacher and child data and specifically integrates FCT, makes a unique contribution.

Similarly, Artman-Meeker et al. (2017) provided FCT instruction to behavior analyst candidates using BIE; however, no child-level outcomes were reported in this study. N. E. Rosenberg et al. (2020) provided on-site instruction to support staff through BIE, aiming to increase children’s self-advocacy behaviors. Previous studies have focused on the effects of BIE on adult learners, with child outcomes evaluated to a limited extent. In contrast, this study evaluated both the accuracy of teacher candidates’ implementation and the increase in communication skills and decrease in problem behaviors in children with autism. While following a similar methodology to previous studies, this study makes a unique contribution to the literature by systematically examining child outcomes. Additionally, since the legal and professional definitions of behavior analysts and paraprofessionals are not established in Turkey, this study contributes significantly to the local literature by exploring how BIE can be implemented in teacher training contexts.

The processes of training special education teachers in Türkiye face various structural limitations in the development of implementation-based competencies. Content related to applied behavior analysis is generally presented at a theoretical level, and PTs are not provided with sufficient opportunities to learn complex intervention programs practically. Furthermore, the lack of legal or professional definitions for roles such as behavior analyst or paraprofessional in Türkiye makes it challenging to implement science-based practices in a systematic and qualified manner in the field. In this context, there is a need for structured, accessible, and sustainable support models to help PTs develop their practical skills in conditions where face-to-face supervision is limited. Technology-supported coaching approaches such as BIE offer a potential solution to this gap by providing an alternative that can be adapted to the existing higher education structure and school practices in Türkiye. Additionally, the limited cultural practice of providing face-to-face critical feedback may make structured feedback processes at the online and individual levels more acceptable and effective for PTs.

Another unique feature of the study was the long-term maintenance data (four sessions and up to seven weeks) for PTs and children. In studies where PTs were coached using BIE, the maintenance data collected for PTs were limited to three sessions, and in only one study (Scheeler et al., 2012), were collected near the end of the intervention (Coogle et al., 2017; Scheeler et al., 2012).

When the social validity findings of the study are analyzed, it is seen that the opinions of the PTs before the intervention changed positively compared to after the intervention. As a result of the intervention, PTs found eCoaching with BIE effective in learning an intervention easily, preferred BIE to face-to-face coaching, preferred immediate feedback to delayed feedback, and found eCoaching with BIE time-saving (M:5). Only one PT remained undecided about the cost of eCoaching offered through BIE at the end of the study. This may be attributed to the fact that the PT had no previous experience with face-to-face coaching. The social validity findings are consistent with other studies using eCoaching with BIE (Artman-Meeker et al., 2017; M. Rock et al., 2012).

In the study, social validity data were collected both before and after the intervention. In recent years, there have been differing views on the importance of collecting social validity data before the research begins to establish the effectiveness of the practices used and to ensure that social validity is evaluated throughout the process. In this study, the collection of social validity data before and after the intervention is important in terms of reflecting teacher candidates’ evaluations of the eCoaching process, both at the expectation level and after the experience. In particular, it has been argued that evaluating social validity not only after the intervention but also throughout the process provides methodologically stronger results. In this study, pre-intervention data collection determined the initial attitudes of PTs toward the process, while post-intervention data revealed changes in their perceptions of the intervention. This two-way data collection approach demonstrates that social validity is systematically addressed not only at the outcome level but also at the process level, thereby increasing the methodological validity of social validity findings. Furthermore, all PT evaluations were collected through five-item structured statements designed to measure social acceptance directly related to the intervention. In this regard, the fact that the social validity assessment was conducted in a structured and temporally controlled manner in the study provides a unique and robust contribution from a methodological perspective.

During the implementation phase, the wide distribution of accuracy rates among some participants may be attributed to PTs’ difficulties in applying newly learned skills, particularly during the first sessions of the intervention. As the coaching process progressed, this variability decreased, and performance accuracy became more stable. This fluctuation was considered a natural part of the learning process, especially for participants with lower initial levels. Therefore, the evaluation was based on general trends and level analyses. In terms of intervention sequence, although a slight upward trend was observed in the baseline data of PT2, this trend was based on a limited number of data points. It did not constitute a consistent upward trend, thus not posing an obstacle to the transition to implementation. Furthermore, the initiation of intervention with PT2 was carried out by logistical planning, class program compatibility, and the coaching schedule. By the basic principles of the multiple assessment design, intervention decisions for each participant were made by jointly evaluating visual analysis and practical conditions.

In the BIE eCoaching literature, some concerns about confidentiality have been mentioned. Maintaining confidentiality and protection from possible cyber attacks in establishing the connection between the coach and the PT has been emphasized (N. Rosenberg & Huntington, 2021). In this study, additional measures were taken to enhance data security. The recorded sessions were saved on a cloud system connected to an encrypted email address. In addition, it was ensured that no third person entered the environment where the participant couple and the coach were present during the sessions. The consent forms also included confidentiality principles (Cavalari et al., 2015).

It is also stated that the disruptions that may arise from using technology in eCoaching practices may affect the flow of the practice (N. Rosenberg & Huntington, 2021). For this reason, some measures suggested by Lee (2005) was taken. Accordingly, it was ensured that the laptops were updated to prevent them from shutting down in the middle of the implementation, that the laptops were properly plugged in, even if they were fully charged before the implementation, and that the Bluetooth headsets were fully charged. Before starting the research, we also checked that the internet connection in both environments was strong enough to maintain an uninterrupted connection (Simple Practice, 2020). Using the Pro version of the Zoom^TM^ application prevented the duration of the session from being limited.

Additionally, a competency analysis was prepared and provided to the PT before the intervention, enabling them to perform the technology setup safely and effectively. These measures did not cause any interruption in any of the sessions due to technological disruptions. In the study, the coach observed the recordings at the end of each session to ensure that the PTs focused on the feedback and addressed this concern. This situation positively affected the learning behaviors of the participant pairs in the study, as they were not distracted due to these reasons. In some studies, concerns have been raised that technology-mediated communication may affect the naturalness of behaviors (N. Rosenberg & Huntington, 2021). It is believed that the absence of these disruptions also contributes to the naturalness of the environment.

### 4.1. Limitations

Although the findings of this study are positive, some limitations should be considered. First, it should be noted that if the teacher misidentifies the function of the problem behavior, the intervention may be ineffective or may even increase the behavior problems. Additionally, it is essential to consider that an individual’s behavior may change over time, which can limit the validity of intervention plans. Therefore, accurate and ongoing assessment of function is critical for the effectiveness of intervention programs such as FCT.

Additionally, conducting a functional analysis independently requires time, knowledge, and technical expertise. Teachers need to be thoroughly trained to carry out this process effectively, which is often not possible in practice. In this context, analyses conducted by licensed or certified behavior analysts may provide more reliable results. However, providing such professional support may impose additional time and resource burdens on institutions. Therefore, access to system-level expert support in school-based interventions is a crucial determinant of their sustainability.

Although the present study aims to provide as natural an environment as possible during the sessions with the children, the process of preparing technological tools for recording and coaching may have slightly reduced the naturalness of the activity. All PTs who participated in this study had a computer/tablet. They also had an internet connection with a strong infrastructure in the homes of children with ASD. The researchers only provided Bluetooth headsets. However, given the available resources in Turkey, not all PTs or children in all regions may have these devices at home. Since these variables will directly affect the study’s implementation and data collection process, they can be considered a limitation in other cases.

Conducting the study in the home environment of children with ASD made it difficult to control external variables. For example, during the implementation, the movements and sounds of other individuals living at home occasionally distracted the participants.

One limitation of this study is related to the social validity measure used. Although social validity data provided essential insights into the perceived usefulness and feasibility of the intervention, the measure itself was not a standardized tool with established reliability and validity. Therefore, the interpretation of social validity findings should be made with caution, and future studies are encouraged to employ validated instruments or develop psychometrically sound tools for assessing social validity.

One of the important limitations of this study is the relatively small sample size. The findings of the research, which was conducted with three teacher candidates and three children, may be specific to the characteristics of the participants and may have limited generalizability to a broader population. Therefore, the results should be interpreted with caution, and further studies with different sample groups should be conducted to support the consistency and generalizability of the findings.

In this study, the coach has performed two roles: implementing coaching and conducting functional analysis. This situation can be considered a limitation.

### 4.2. Future Research

This study has provided evidence of the effectiveness of real-time performance feedback provided to special education PTs through BIE eCoaching. However, future research should employ comparative designs (e.g., randomized assignment or multiple baseline designs) to more clearly elucidate the effects of feedback timing (immediate vs. delayed) on instructional outcomes. Such methodological rigor will provide more unmistakable evidence of the effects of feedback timing on implementation accuracy, persistence, and generalization.

Furthermore, while the present study examined both PT and child outcomes, many studies in the BIE literature have focused solely on teacher behavior. Future studies employing double-dependent variable designs that measure both teacher implementation accuracy and child behavior change simultaneously will enable stronger conclusions about the effectiveness of the intervention. Repeated collection of social validity measures for children throughout the process is also valuable for revealing how meaningful the intervention is from the learner’s perspective.

In addition, to increase the level of real-life relevance (ecological validity) and generalizability of the interventions, it is recommended that future research test BIE interventions on different groups of practitioners (e.g., classroom teachers, support staff, family members). Comparing the effects based on the type of technological devices used (mobile devices vs. computers) can also be evaluated through adapted alternative treatment designs or cost-effectiveness analyses. Furthermore, including structured generalization sessions and conducting long-term follow-ups will strengthen claims regarding the sustainability of BIE-supported implementation.

## 5. Conclusions

The findings of this study indicate that BIE eCoaching is an effective method for special education PTs to implement functional communication instruction with high implementation fidelity. This result provides a sustainable and accessible support model for contexts where face-to-face supervision is limited in teacher training processes. Additionally, PTs’ ability to retain and generalize the skills they learned provides strong evidence of the long-term effectiveness of BIE eCoaching. The reduction in problem behaviors and increase in communication attempts observed in students suggest that this approach can yield indirect yet meaningful outcomes for children. These findings not only demonstrate the effectiveness of the method but also offer insights into how the gap between research and practice can be bridged. Therefore, this study constitutes an essential contribution to teacher training, the delivery of special education services, and the implementation of effective remote support programs.

## Figures and Tables

**Table 1:** Social validity findings obtained from pre-service teachers.

Items	Pre-Intervention				Post Intervention			
	PT1	PT2	PT3	M	PT1	PT2	PT3	M
With BIE coaching, I can easily learn to use an intervention.	2	3	2	2.3	5	5	5	5
I prefer BIE coaching to face-to-face coaching.	1	2	1	1.3	5	5	5	5
I prefer immediate feedback to delayed feedback during implementation.	3	3	3	3	5	5	5	5
I think BIE coaching is less costly than face-to-face coaching.	3	3	3	3	5	4	3	4
I think BIE coaching will take less time than face-to-face coaching.	2	3	3	2.6	5	5	5	5
M	2.2	2.8	2.4		5	4.8	4.6	

Social validity was scored on a scale of 1 (strongly disagree) to 4 (strongly agree).

## Data Availability

The data will be shared with the editorial office upon request.

## Abbreviations

The following abbreviations are used in this manuscript:

ASD	Autism spectrum disorder
PT	Pre-service teacher
FCT	Functional communication training
BIE	Bug-in-ear
FAOB	Functional analysis of other behaviors
C	Children

## Appendix A

**FCT Implementation Fidelity Data Recording Form_sessions consisting of trials with 0 s of delay time**					
**Name:**					
**Target Behavior:**					
**Child’s Name:**					
**Date: …/…/…**					
Trials in which the prompt is presented immediately after the stimulus	1	2	3	4	5
1. Allow the child to engage with their preferred object/food/activity for a period of time (e.g., 3–5 s with food or drink, 20–30 s with an object or activity).					
2. After a period of time (e.g., 3–5 s for food or drink, 20–30 s for an object or activity), take the child’s preferred object/food/activity away from them.					
3. Without waiting for the child’s behavior, provide a controlling prompt (e.g., verbal, physical, modeling). Wait 4 s for your child to respond.					
4. When the child demonstrates the targeted communication skill (e.g., saying the name of the food/object/activity) using the prompt you provided, say the name of the food/object/activity and reinforce the child and allow the child to interact with the desired object/food/activity.					
5. Ignore any problem behaviors the child exhibits.					
6. When the child demonstrates the targeted communication skill using the prompt you provided, place a “+” sign in the behavior column following the prompt in the relevant row of the data recording form related to communication skills. When the child exhibits a behavior different from the targeted communication skill or does not exhibit any behavior, place a “−” sign in the behavior column following the prompt in the relevant row of the data recording form related to the communication skill. When the child exhibits problem behavior, place a “+” sign opposite the relevant row associated with the problem behavior.					
7. After the child has engaged with the object/food/activity for a period of time (e.g., 3–5 s with food or drink, 20–30 s with an object or activity), take the object/food/activity from the child’s hand to move on to the next trial.					
**Total Number and Percentage:**					
**Steps with Feedback Provided:**					
**FCT Implementation Fidelity Data Recording Form, sessions consisting of trials with 4-s of delay time**					
**Name:**					
**Target Behavior:**					
**Child’s Name:**					
**Date: …/…/…**					
After the target stimulus, wait 4 s and then present the prompt.	1	2	3	4	5
1. Allow the child to engage with their preferred object/food/activity for a period of time (e.g., 3–5 s with food or drink, 20–30 s with an object or activity).					
2. After a period of time (e.g., 3–5 s for food or drink, 20–30 s for an object or activity), take the child’s preferred object/food/activity away from them.					
3. Wait 4 s for the child to demonstrate the targeted communication skill.					
4. When the child demonstrates the targeted communication skill (e.g., saying the name of the food/object/activity) within 4 s, say the name of the food/object/activity and reinforce your child (e.g., “Good job. You did a great job”.) and allow the child to engage with the desired object/food/activity.					
5. Ignore any problem behaviors the child exhibits.					
6. When the child demonstrates the targeted communication skill within 4 s, place a “+” sign in the behavior column before the hint related to communication skills in the data recording form. When the child exhibits problem behavior within 4 s, place a “+” sign opposite the relevant row related to problem behavior.					
7. After the child has been engaged with the object/food/activity for a period of time (e.g., 3–5 s with food or drink, 20–30 s with an object or activity), take the object/food/activity away from the child’s hand to move on to the next trial.					
8. If the child (a) does not exhibit any behavior within 4 s, (b) exhibits behavior other than the targeted communication skill, or (c) exhibits problem behavior, provide a controlling prompt (e.g., verbal, physical, modeling) at the end of 4 s. Wait 4 s for the child to respond.					
9. Ignore any problem behaviors the child exhibits.					
10. When the child demonstrates appropriate communication skills (e.g., saying the name of the food/object/activity) with the hint you give, say the name of the food/object/activity and reinforce the child (e.g., “Good job. You asked very nicely”.), allow the child to engage with the object/food/activity they requested.					
11. If the child exhibits behavior different from the targeted communication skill within 4 s before the prompt, or does not exhibit any behavior, place a “−” mark in the behavior column before the cue in the relevant row of the data recording form. When the child exhibits the targeted communication skill after the cue, place a “+” sign in the “behavior after the prompt” column of the relevant row in the data recording form. When the child exhibits a behavior different from the targeted communication skill after the prompt is presented, or does not exhibit any behavior, place a “−” sign in the behavior column following the prompt in the relevant row of the data recording form related to communication skills. When the child exhibits problem behavior within 4 s, place a “+” sign opposite the relevant row related to problem behavior.					
12. After the child has engaged with the object/food/activity for a period of time (e.g., 3–5 s with food or drink, 20–30 s with an object or activity), take the object/food/activity away from the child’s hand to move on to the next trial.					
**Total Number and Percentage:**					
**Steps with Feedback Provided:**					
